# A Pleased Patient

**Published:** 1904-12

**Authors:** 


### A PLEASED PATIENT.

There's not much comfort in a name. Your patient doesn't care whether it's a cavity, an exposed nerve, or an ulcerated tooth. If there's pain, that's enough for the patient. And if there are Antikamnia and Codeine Tablets, that's enough for the dentist. Pain is often all there is to it. Stop the pain and you please the patient. Dentists when operating should administer one Antikamnia and Codeine Tablet every hour, giving one shortly before beginning the operation. One tablet before and one after extracting a tooth, will prevent the severe pain and consequent nervousness. Facial neuralgia, toothache and earache can be easily relieved with one tablet every hour or two.

